# Influenza in Horses*Read before the Border Counties Veterinary Medical Society.

**Published:** 1891-03

**Authors:** George Fleming


### INFLUENZA IN HORSES.*

BY DR. GEORGE FLEMING.

Of the few diseases about which there is now anything approaching the mystery that not long ago enshrouded so many epizoötic and epidemic disorders, influenza probably holds the foremost place, so far as obscurity of origin, cause of diffusion, or mode of generalization are concerned.

As I have shown in my work on "Animal Plagues," this malady—or one more or less closely resembling it—has been known for many centuries; attacking at one time the human species only, at another time the equine species, and in some outbreaks both species at the same period, or one species shortly before the other.

In tracing its history I have been able to discover a serious and wide-spread disease among horses in Yemen, Arabia, so long ago as A. D. 1328 ; this was more of a bilious fever, however, if one may hazard a guess from the symptoms described. But, in reality, the more extensive invasions have only been recorded for about two hundred years and, as I have mentioned, some of these have coincided with influenza in man. In the last century records there is some allusion to these coincidences. Huzard, for instance, speaks of influenza attacking horses in the spring of 1776, after the human species had been affected. And in this century it so appeared in 1803, 1805, 1833, and 1858.

But by far the largest number of outbreaks among horses occurred without mankind being in any way involved.

Some of the influenza epizoöties have been very remarkable for their wide and rapid diffusion, but I cannot refer to these now without encroaching too much on the time and patience of the meeting ; so those who are curious in the matter of the history

* Read before the Border Counties Veterinary Medical Society.

of the malady are referred to my volumes on "Animal Plagues." I may make mention, however, of the remarkable epizoöty of catarrhal influenza that occurred throughout Germany, Bohemia, and Moravia in 1746; and to the perhaps still more extraordinary one, and certainly the best recorded, that appeared on the American Continent in 1872–73. This outbreak seems to have commenced in Toronto, Canada, in September, 1872, and radiating thence in every direction, it visited all the cities and towns in Canada, and spread over the United States as far south as Virginia, and westernly as far as Chicago. Within two months it had invaded the States and Territories of the far West; it spread over California and, travelling onward, at last reached British Columbia, Cuba, Mexico, and Central America.

As has been remarked, perhaps no other disease with which we are acquainted commences more secretly and suddenly, extends more rapidly, or tends more readily to become a world-wide malady, than this; and notwithstanding our recent experience of it in mankind and horses, it must be confessed that there is very much in relation to its genesis and propagation, as well as to its immediate exciting cause, which still remains for elucidation. I cannot hope to throw any new light upon these obscure points in the pathogeny of the disease; but I will endeavor, to the best of my ability, to bring our knowledge up to date, not only with regard to these points, but also with respect to the sanitary and medical treatment of the malady.

In the first place, then, the question might be asked, What is influenza?

In the human and equine species the term has been applied to an intense febrile condition, occurring suddenly in perhaps a sporadic form for a brief period, then extending rapidly and affecting a large number of individuals—the majority in a village, town, city, country, continent, or even several continents being attacked; and this irrespective of situation, climate, season, surroundings, and even in defiance of all sanitary measures except rigid isolation. So far the malady has the same character in man and the horse. And in the symptomatology, to some extent, there is a close resemblance, if not absolute identity.

In the old descriptions of the outbreaks of influenza the disease appeared as a severe catarrhal fever, accompanied by great depression and sickness, and running its course rapidly. We do not find much mention of complications, or of any special symptoms predominating in different invasions, except those of catarrh.

But whether these were not observed, or were not present, or whether we have now more than one disease, or a disorder somewhat multiform, certain it is that at one time we have among horses a widely prevalent affection, characterized by certain marked features; at another time, another outbreak as general, in which these are either absent, or masked by characters still more striking; and, again, in another epizoöty we shall have symptoms that would almost lead to the belief that we have quite another disorder to deal with. These differences are not marked in individual cases in the same outbreak, but constitute general characteristics of different invasions.

Notwithstanding these diverse manifestations, there are certain features which are always present in all the outbreaks, and which compel us to cling to the old and vague term, influenza. These are the abrupt onset of the malady—the very sudden transition from vigorous health to intense prostration—from normal temperature to extreme hyperpyrexia—from activity and gaiety to immobility and profound depression in a few hours. In no disease with which I am acquainted does the temperature rise so high and so rapidly, and the physiognomy of the disorder is generally so striking, that the veriest tyro could not mistake it for any other disease. The drooping head and ears, listless look, swolled, tearful eyes, and general facial expression, betray cephalalgia, debility, and nausea; while the hot, dry skin, the pasty, burning mouth, the internal temperature reaching, perhaps, 106° or 107° Fahr., and the almost suppressed secretions—all testify to the remarkable pyrexial condition of the animal, which is still further accentuated by the pulse and respirations; at the same time the disinclination to move—the desire to remain fixed in one spot, and, if compelled to stir, the groans and stiffness—betray muscular soreness in loins and limbs.

Another feature is the rapid loss of condition—a fat animal soon becoming much reduced in bulk; but this change might be anticipated when the very abnormal high temperature—synonymous with greatly increased tissue oxidation—is considered; and the extraordinary drop in the temperature that ensues when convalesence sets in is also to be noted. I have found it in some instances as low as 97° degrees Fahr.—the normal temperature of the horse being estimated at 100°.

Whether we have one disease presenting itself in different forms at different outbreaks, or whether different diseases are included in the designation of influenza, these general symptoms are always present in a more or less degree, and constitute the special features of the epizoöty.

If we assume that there is only one influenza, then we must take cognizance of its different forms, and in this regard—and notwithstanding its offering itself as a somewhat bewildering multiform disorder—it may be accepted that it shows itself at different oubreaks in either of three forms, which are suficiently distinct; though there may be complications, or accidental epiphenomena, to obscure them in individual cases.

*First Form.*—The simplest form is the catarrhal, in which the mucus membranes of the head are chiefly involved, and, consecutively, those of the deeper air-passages, often leading to capillary bronchitis and broncho-pneumonia under unfavorable conditions. This is perhaps the most common form of influenza, and the one which is attended with least mortality; though the general symptoms I have already alluded to may be present in their greatest development. This is the form, also, with which we are so familiar in mankind; and it is that which, both in man and the horse, becomes at times of world-wide prevalence.

But it may here be necessary to add that, however closely allied the influenza of the horse and that of mankind may be in their origin, mode of extension, and symptomatology, yet we cannot admit their absolute identity; inasmuch as the influenza of man cannot be conveyed to the horse, nor that of the latter to the human species. There is not a well-authenticated instance on record of such transmission having occurred, notwithstanding the comparative frequency of outbreaks of the disease, even within my own recollection. If such transmission were possible—at any rate, from horse to man—it must have been observed in our mounted corps in the army, where such close attention is paid to sanitary matters, and where the men are always in such close contact with their horses, whether these be well or ill.

Not only this, but the greatest outbreaks of influenza in man have not been coincident with those in the horse. When the human populations of countries far and wide have been prostrated and panic-stricken by the mysterious invasion, horses remained unaffected; and when the majority of the horses in towns and cities have been incapable of leaving their stables, because of being under the sway of their influenza, their immediate attendants, as well as the inhabitants of these towns and cities, were unaffected. Every outbreak in this country furnishes evidence of the fact that the human and equine influenza are not due to the same, but to closely allied exciting causes, and that the factor or factors which produce the disease in the one species will not do so in the other. No better, because, perhaps, no stronger, proof of this could be adduced than that yielded by the great influenza epizoöty of the American continent in 1872–73, when all horse traffic was for a while suspended, and the streets of the large cities were comparatively silent, because of the plague-stricken condition of the horses. And yet there was not a case of influenza recorded among the people. It was somewhat the same during the present year, when human influenza had spread to nearly every part of the world, and horses remained free from it.

It is somewhat curious that horses and mankind should be the only creatures to suffer from influenza. The history of animal plagues shows that dogs and cats have, at rare intervals, been attacked by an epizoötic catarrhal fever; but these happened long ago, and though horses and mankind have been frequently visited by influenza, yet dogs and cats have remained immune, and there is not an instance known of their having been infected from man or horse.

This catarrhal form of influenza is that which becomes most generally and most widely diffused—in serious outbreaks as many as 80 or 90 per cent. of the horses becoming affected; though the mortality may not be greater than from 2 to 4 per cent. of those attacked, and convalesence is rapidly established unless there are grave complications.

*Second Form.*—The second form differs from the other in that, besides the general intensely febrile and adynamic symptoms which mark influenza in its several forms, the chest is chiefly involved—in fact, the disease might be termed epizoötic pleuro-pneumonia of equines. It differs from the other form in being more insidious in its invasion, less wide-spread, in that it does not invade entire countries, but is limited to certain towns, portions of towns, and even certain stables or barracks. Indeed, it would almost appear to be enzoötic; for we have a barrack in London, in which a regiment of the Household Cavalry is located—Regent's Park Barracks—in which almost every year there is an outbreak. Rarely witnessed in stables containing only a few horses, this form appears where large numbers are kept, and it is perhaps the most troublesome form to contend with, as it is attended by the largest mortality, and is followed by the most protracted convalescence. Sanitary arrangements did not seem to have much influence on its extension, as it manifests its presence in good stables as well as bad ; though there must be some local influence at work to account for its erratic course, distribution, and persistency in some places, while others remain exempt—though adjoining those infected—or the disease soon disappears if it does invade them. Catarrhal symptoms may be entirely absent, a very slight nasal discharge of a transparent yellowish fluid only being noted. Almost from the commencement there are indications that the lungs are extensively involved ; and yet so intense is the debility that—contrary to what usually happens in sporadic pneumonia—a stricken animal often lies down—particularly towards the end of the attack, when dissolution is impending. A septic or gangrenous condition of the lungs is indicated—even before death occurs—by the odor of the exhaled air from the nostrils, and the putrid blood discharges from the same openings ; indeed, it is not unusual to find, on *post mortem* examination, that there are local hemorrhages into various tissues and organs, and general appearances which would lead to the supposition that the horse had succumbed to septicæmia.

*Third Form.*—This form is like the last in so far that it is erratic and limited in its spread, appearing only here and there in towns, and in being localized in districts or counties. But it differs from it in the skin and subcutaneous tissues being mainly involved—in fact, the disease might be considered as erysipelatous. The head and limbs are the parts chiefly attacked, and sometimes these attain a great size ; the lower surface of the chest and abdomen also frequently share in the tumefaction. The digestive organs are implicated in some outbreaks of this form of influenza ; their mucus membrane, and also that lining the nasal passages being congested, softened, ecchymosed, and in severe cases sloughing in patches. In this form convalescence is somewhat long, but the mortality is not very great when proper treatment is adopted. These are the forms which, I think, we may assert that influenza assumes. Of course they may be modified in individual cases by circumstances ; but their general features will be recognized by those who have had a considerable experience of the disease.

With regard to the cause of influenza, I do not hesitate to class it with those maladies which are produced by microbes ; and this notwithstanding the fact that no special organism has yet been fixed upon as the influenza germ. But that it will be isolated, examined, cultivated, and its life history studied, I entertain no doubt whatever ; indeed, it may happen that we shall be able to utilize it by compelling it to provide an antidote for its bane. The development, course, and symptoms of the malady point out in the clearest manner that it is of microbic origin ; though whether the three forms I have referred to are due to one and the same germ is extremely doubtful.

The erysipelatous form reminds one very much of one of the forms of anthrax met with among horses in India, in which external swellings are a special feature. Indeed, the Austrian veterinary surgeons are inclined to regard this form of influenza as anthracoid in its nature ; and they base their opinion on the fact that they have found anthrax bacilli in the exudations and tissues of sick animals. But anthrax, as we see it in India, is much more rapid in its course and fatal in its termination than this form of influenza. However there is no reason why the microbe producing it should not be a modification of the *Bacillus anthracis*, modified by climate and other conditions. At any rate, it appears to be proved that the pectoral form of influenza of the horse is due to diplobacteria ; and the analogy of the disease to septicæmia is further strengthened by the circumstance that, when these bacteria are inoculated into mice, these rapidly die of septicæmia.

The catarrhal form of influenza may be due to another kind of micro-organism—probably the pneumoccocus or the bacillus of Friedlander.

Climate and temperature do not appear to have any influence in its production. It prevailes in the most diverse climates and at all temperatures and the closest and most extensive and recent observations show that it does not spread by virtue of any of the recognized conditions of cold, heat, humidity, season, climate, or altitude. In the epizoöty of the American continent it prevailed and was propagated in the cold of a northern winter, and in the summer heat of Central America ; in the dry air of Minnesota, and in the moist air of the seaboard ; at an altitude of 5,000 feet above the sea (at Saltillo, Mexico), and on the low levels of New Orleans (10 feet above sea level). And it is certainly not disseminated by winds, but often spreads against them.

The notion that influenza is due to an excess of ozone, or the presence of any gaseous or material product in the atmosphere, cannot be entertained nowadays. If such were the case, many other species of creatures besides man and the horse would suffer at the same time. This does not seem to have struck those who, for so many years, have tried to account for the prevalence and extension of the disorder on this hypothesis.

That influenza is infectious I personally have no doubt, though it may be extended in other ways than by immediate contact of sick with healthy horses I am not prepared to deny. The proofs in favor of its infectiousness in horses support my opinion, and this opinion I have entertained for very many years, as will be seen by a reference to my work on "Veterinary Sanitary Science and Police." In the American outbreak of 1872–73, for instance, it commenced at Toronto, and spread thence as from a centre, no locality being exempt which was known to have been in communication by means of horses or mules with places in which the disease existed; and those places which were not visited by it were so situated that the introduction of horses or mules was in some of them impossible, and in others of them improbable. Another proof which I rely upon is that equine influenza has not, so far as I can ascertain, ever been witnessed in Australasia, South Africa, India, or New Zealand, and this because, as I view it, it has never been carried there. The microbe which causes it in horses has no action on other creatures; and it may be taken for granted that, once it obtains access to the body of a susceptible animal, it can multiply in a wonderful manner, in order to produce those chemical changes in the fluids which enable them to act on the organism as if most active septic matter had been introduced into it.

I may call attention to the fact that the ass, which is so nearly allied to the horse, appears to be exempt from influenza; for I have never met with or heard of its occurrence in that animal, and even mules do not appear to be very susceptible.

Of the influences which are at work in exciting the activity of the microbe at certain periods we know nothing. We may assume that it can multiply without as well as within the body, like the germ of miasmatic diseases, and that certain atmospheric or telluric conditions favor its development and multiplication to an astonishing degree. Though we are in ignorance of those conditions which favor the growth and spread of this particular microbe—the existence of which we can only assume for the present—yet it must be confessed that we are no wiser with regard to the genesis and advent of creatures which are well known, and which are also injurious to the interests of mankind. Take the immense swarms of locusts which in some parts of the world, at uncertain periods, sweep over great surfaces of the earth, consuming everything in the nature of vegetation which they meet with on their long flight; or the countless swarms of field-mice or similar vermin, which spring up now and again, in certain countries, from nobody knows where, and devastate great tracts. Why should similar influences not operate in the evolution of myriads of influenza-exciting micro-organisms ?

But experience demonstrates that, if the microbe can multiply outside the body, it can also multiply within it, and that therefore a diseased animal can infect healthy ones ; and there can scarcely be a doubt that infection is a potent factor in the spread of the malady.

With regard to the treatment of the disease, I confess to having little faith in the administration of drugs, but rely more on good nursing, cleanliness, plenty of fresh air, ample space, and comfort.

Medicines which act as germicides, when they can be administered with safety, may be given. Those which operate in reducing the fever generally act in this way. For many years I relied solely on preparations of quinine, and have reason to believe that it has an almost specific effect on influenza. Preparations of carbolic acid are, of course, much less expensive, and might have a similar effect ; so might antipyrin, and other antifebrile remedies. But I need not dilate on the treatment of the disease, as I have no doubt you are all perfectly well aware of the necessity for dealing with symptoms as they arise ; and as these vary more or less in nearly every case, close watchfulness and keen judgment are needed, and not rule-of-thumb practice.

